# Supplementary material for: A selective role for ventromedial subthalamic nucleus in inhibitory control
Source: eLife. 2017 Dec 4;6:e31627. doi: 10.7554/eLife.31627 (PMC5730370; doi:10.7554/eLife.31627)
Supplement: Supplementary file 1. [file elife-31627-supp1.docx]

Supplementary file 1. Number of STN neurons found in each category per monkey

|  | **monkey C** | **monkey H** | **Total** |
| --- | --- | --- | --- |
| **Recorded cells** | 55 | 112 | 167 |
| **Movement cells** | 34% (19/55) | 27% (30/112) | 29% (49/167) |
| **Switch-Stop cells** | 14% (9/55) | 5% (5/112) | 8% (14/167) |
| ***Stop cells*** | 6 | 2 | 8 |
| ***Switch cells*** | 3 | 3 | 6 |
| **Switch-Go cells** | 18% (15/55) | 23% (26/112) | 25% (41/167) |
| ***Increased activity*** | 13 | 20 | 33 |
| ***Decreased activity*** | 2 | 6 | 8 |
| **Torque cells** | 29% (16/55) | 23% (26/112) | 25% (42/167) |
| **Go cells** | 42% (23/55) | 34% (38/112) | 37% (61/167) |
| **NoGo cells** | 25% (14/55) | 24% (27/112) | 25% (41/167) |
| **Error-related cells** | 11% (6/55) | 10% (11/112) | 10% (17/167) |
| **Reward-related cells** | 5% (3/55) | 4% (5/112) | 5% (8/167) |
