# Supplementary figures and images for: A selective role for ventromedial subthalamic nucleus in inhibitory control
Source: eLife. 2017 Dec 4;6:e31627. doi: 10.7554/eLife.31627 (PMC5730370; doi:10.7554/eLife.31627)

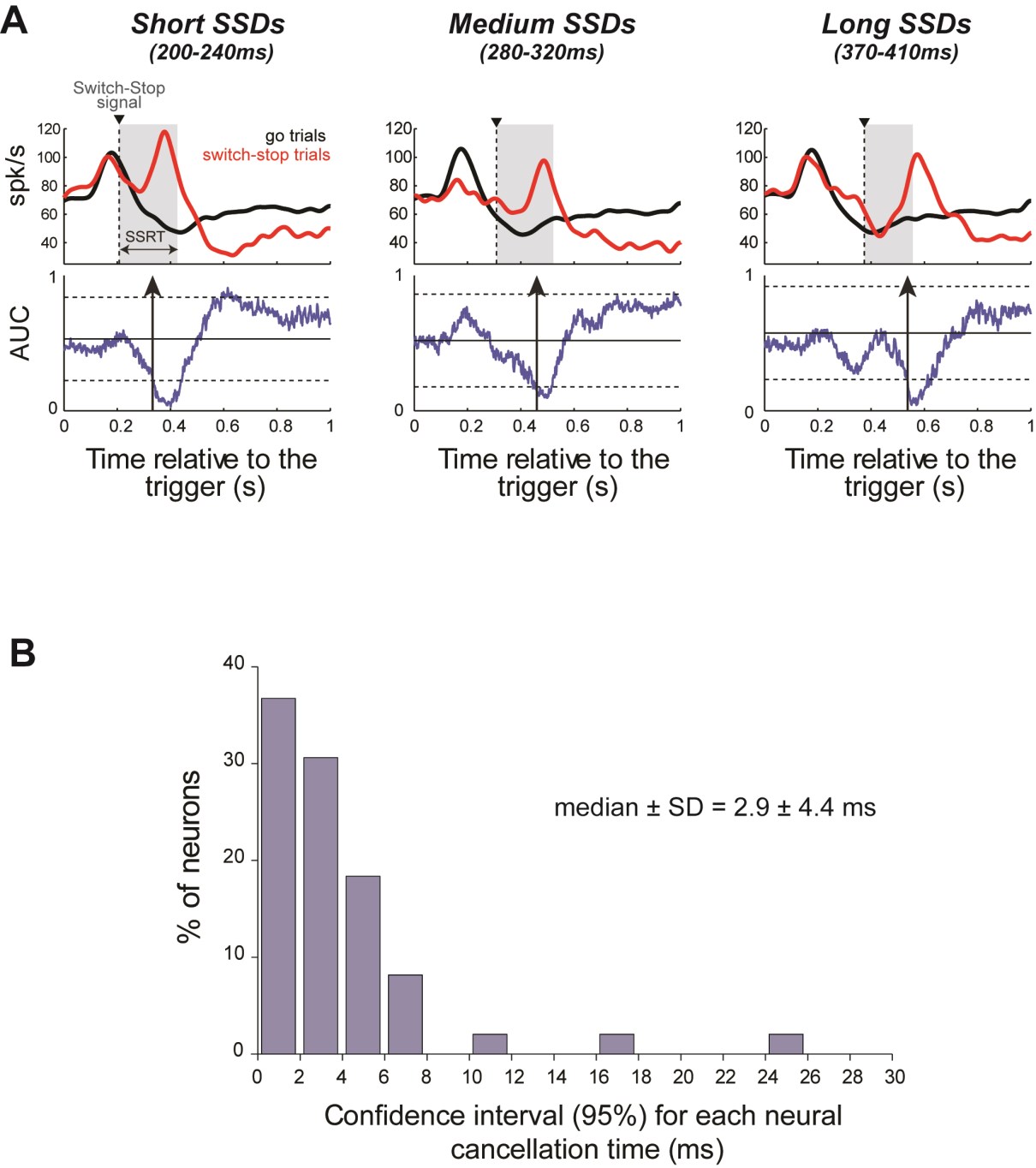

Supplement: Supplementary file 2. — (A-B) Population-averaged activities of (A) Go cells and (B) NoGo cells that showed a short-latency response (<300 ms) to the Switch-go signal. Spike density functions aligned on switch-go signal presentation (green) and the equivalent time in no-go trials (gray) were normalized by subtracting the baseline activity (500 ms before the signal) and grouped according to the response pattern evoked in neuronal activity: increase (left) or decrease (right) in firing relative to no-go trials. The vertical width of the spike density function line indicates the population SEM. To compare the firing rate between trials, we used the same ROC analysis (p<0.01). The arrows in AUC values indicate the times of first differential activity. (C) Distribution of peak AUC values for the Go/NoGo comparison plotted versus the AUC values for the switch-go comparison. No relationship was observed in the STN between both reactive and proactive encodings (χ2=1.14, p=0.29). [file elife-31627-supp2.docx]
